# Genetic polymorphisms associated with the inflammatory response in bacterial meningitis

**DOI:** 10.1186/s12881-015-0218-6

**Published:** 2015-08-28

**Authors:** Fabrícia Lima Fontes, Luíza Ferreira de Araújo, Leonam Gomes Coutinho, Stephen L. Leib, Lucymara Fassarella Agnez-Lima

**Affiliations:** 1 Departamento de Biologia Celular e Genética, Universidade Federal do Rio Grande do Norte, UFRN, Natal, Brazil; 2 Institute for Infectious Diseases, University of Bern, Friedbuehlstrasse 51, CH-3010 Bern, Switzerland; 3 Departamento de Biologia Celular e Genética, Centro de Biociências – UFRN, Campus Universitário, Lagoa Nova, Natal, RN 59078-970 Brazil

## Abstract

**Background:**

Bacterial meningitis (BM) is an infectious disease that results in high mortality and morbidity. Despite efficacious antibiotic therapy, neurological sequelae are often observed in patients after disease. Currently, the main challenge in BM treatment is to develop adjuvant therapies that reduce the occurrence of sequelae. In recent papers published by our group, we described the associations between the single nucleotide polymorphisms (SNPs) *AADAT* +401C > T, *APEX1* Asn148Glu*, OGG1* Ser326Cys and *PARP1* Val762Ala and BM. In this study, we analyzed the associations between the SNPs *TNF* -308G > A*, TNF* -857C > T*, IL-8* -251A > T and BM and investigated gene-gene interactions, including the SNPs that we published previously.

**Methods:**

The study was conducted with 54 BM patients and 110 healthy volunteers (as the control group). The genotypes were investigated via primer-introduced restriction analysis-polymerase chain reaction (PIRA-PCR) or polymerase chain reaction-based restriction fragment length polymorphism (PCR-RFLP) analysis. Allelic and genotypic frequencies were also associated with cytokine and chemokine levels, as measured with the x-MAP method, and cell counts. We analyzed gene-gene interactions among SNPs using the generalized multifactor dimensionality reduction (GMDR) method.

**Results:**

We did not find significant association between the SNPs *TNF* -857C > T and *IL-8* -251A > T and the disease. However, a higher frequency of the variant allele *TNF* -308A was observed in the control group, associated with changes in cytokine levels compared to individuals with wild type genotypes, suggesting a possible protective role. In addition, combined inter-gene interaction analysis indicated a significant association between certain genotypes and BM, mainly involving the alleles *APEX1* 148Glu, *IL8* -251 T and *AADAT* +401 T. These genotypic combinations were shown to affect cyto/chemokine levels and cell counts in CSF samples from BM patients.

**Conclusions:**

In conclusion, this study revealed a significant association between genetic variability and altered inflammatory responses, involving important pathways that are activated during BM. This knowledge may be useful for a better understanding of BM pathogenesis and the development of new therapeutic approaches.

**Electronic supplementary material:**

The online version of this article (doi:10.1186/s12881-015-0218-6) contains supplementary material, which is available to authorized users.

## Background

Despite immunization programs and effective antimicrobial therapies against bacterial meningitis (BM), the incidences of mortality and neurological sequelae caused by this disease remain high [1]. A complex series of events involving host cytokines, chemokines, proteolytic enzymes and oxidizing agents appear to be responsible for the occurrence of brain damage during BM. This response depends not only on the type and intensity of the stimulation but also on host genetic factors, such as genetic polymorphisms located in coding or regulatory regions of key genes [2–4].

BM is characterized by an acute inflammatory response that is initiated by the presence of bacterial pathogens in the central nervous system (CNS). The disease begins with nasopharyngeal colonization by the pathogen and subsequent invasion of the bloodstream. During this phase, BM can be avoided with efficient innate and acquired immune responses [2, 4].

Several bacterial species that are pathogenic to humans have the potential to cause meningitis, but a relatively small number of pathogens, as *Haemophilus influenzae type b* [Hib], *Streptococcus pneumoniae* and *Neisseria meningitides,* are responsible for the majority of acute BM cases [5, 6]. These primary pathogens utilize distinct but overlapping sets of Toll-like receptors (TLRs) to trigger the inflammatory response, inducing NF-κB activation in a MyD88-dependent pathway [7–9]. Corroborating these data, in a recent study published by our group, we observed a similar profile of cytokine expression during pneumococcal and meningococcal meningitis [10]. Tumor necrosis factor (TNF-α), IL-1β and IL-6 are the major early response cytokines that trigger a cascade of inflammatory mediators, including other cytokines, chemotactic cytokines (chemokines) such as CXCL8/IL-8, MIP-1α/CCL3, MIP-1β/CCL4, MCP-1/CCL2, prostaglandins, matrix metalloproteinases (MMPs), reactive oxygen species (ROS) and reactive nitrogen intermediates (RNI) [2, 9, 11].

In previous studies, we described the associations between BM and the single nucleotide polymorphisms (SNPs) *AADAT* +401C > T (rs1480544) [12], *APEX1* Asn148Glu (rs1130409), *OGG1* Ser326Cys (rs1052133) and *PARP1* Val762Ala (rs1136410) [13]. The *AADAT* gene encodes the enzyme kynurenine aminotransferase II, which is involved in the kynurenine (KYN) pathway, the major route for tryptophan degradation in the brain. Alterations in this pathway are associated with several diseases that compromise the CNS [14]. *APEX1*, *OGG1* and *PARP*1 encode DNA repair enzymes involved in the base excision repair (BER) pathway, one of the most important pathways for the repair of oxidized DNA damage in neurons [15].

In this study, we analyzed the associations between the SNPs *TNF* -308G > A (rs1800629), *TNF* -857C > T (rs1799724), and *IL8* -251A > T (rs4073) and BM. In addition, we also investigated gene-gene interactions, including the SNPs previously investigated by our group [12, 13]. The SNPs *TNF* -308G > A and *TNF* - 857C > T are localized in the regulatory region of the *TNF* promoter and were described to be involved in increased transcriptional activity of the *TNF* gene [16–18]. CXCL8/IL-8 is involved in the chemotactic activity of polymorphonuclear and mononuclear cells. The genetic polymorphisms *IL8* -251A > T causes decreased expression of this chemokine [19–21].

Although several molecular markers have become areas of focus for research, few studies have considered the interplay between markers. In this study, we investigated genetic association data from a model for susceptibility to BM that integrated important routes that are activated during the disease. Our findings demonstrated a higher frequency of variant allele *TNF* -308A in the control group, suggesting a possible protective role against disease, and genetic combinations, primarily involving the alleles *TNF* -308G, *APEX1* 148Glu, *IL8* -251T and *AADAT* +401T, that were associated with the occurrence of BM.

## Methods

### Ethics statement

This work was approved by the Brazilian Ethics Committee (CONEP, CAAE 0052.1.051.000-05). All subjects signed the informed consent agreeing to participate in this research. In the case of minors/child participants involved in this study, we obtained informed written consent from the legal guardians.

### Study population and samples

The study was conducted with a group of 54 patients (35 men and 19 women), 21 individuals were 18 years or younger, 25 aged from 18 to 60 years, 4 individuals aged over 60 years, and 4 with unknown age. The patients were admitted in the Hospital Giselda Trigueiro (HGT), Natal-RN, Brazil, which is a reference hospital for infectious diseases in the state. BM diagnosis was based on the hospital routine, which includes: (1) positive CSF bacterial culture, (2) detection of the pathogen in the CSF by Gram staining plus clinical signs (acute onset, fever, meningeal irritation) and/or (3) positive blood culture or Gram stain in the presence of clinical signs of meningitis, (4) positive bacterial antigen detection in the CSF or blood using the latex agglutination test, with clinical signs of meningitis, (5) CSF parameters including increased protein content (>40 mg/dL), reduced glucose levels (<40 mg/dL) and the presence of CSF pleocytosis (≥500 cells/mm3), with predominantly polymorphonuclear granulocytes (PMN), and (6) symptoms such as high fever, chills, severe headache, nausea and vomiting, and the Kernig and Brudzinski signs of meningo-radicular syndrome. However, some patients with clinical manifestations of bacterial meningitis were on antibiotic treatment prior to definitive diagnosis, so it was not possible to define accurately the etiologic agent. These patients were used only for genotypes analysis and excluded from the inflammatory markers analysis. Moreover, patients undergoing chronic treatment with anti-inflammatory or with other diseases (such as AIDS) that affect the immune and inflammatory responses (e.g., cytokine expression) were excluded from the study. Healthy volunteers and patients attended at the HGT, who had a negative diagnosis for infectious disease, were included in the study as part of the control group, a total of 110 samples (53 men and 57 women), 10 individuals were 18 years or younger, 99 individuals aged from 18 to 60 years and 1 individuals aged over 60 years. These patients were used as controls given that no infection was confirmed and all parameters for diagnosis were normal. In healthy volunteers, CSF samples were not collected, as lumbar puncture is a very invasive method, but a questionnaire evaluating the history of infectious and inflammatory diseases was administered to the healthy volunteers. In addition, blood samples were submitted to obtain white blood cell counts (WBC) and differentials, cytokines and chemokines were analyzed, and it was determined whether the individual had a fever at the time of collection of the biological sample. In relation to BM, 54 patients had a positive diagnosis: 17 were diagnosed with *S. pneumonia*; 7 with *N. meningitides*; 6 with other pathogens; and 24 without a specified etiology. CSF biochemical parameters of each causative agent are shown in Table 1 (more detail can be found in [10] and [22]).

**Table 1:** CSF biochemical parameters of BM meningitis etiologies

CSF (n samples)	Age (years)	Cell count (cells/mm^3^)	Protein (mg/dL)	Glucose (mg/dl)
*S. pneumonia* (17)	27 (11; 43)	1,960 (698;8 3,730)	198.00 (137.50; 339,00)	17 (4; 26.70)
*N. meningitides* (7)	21 (6; 27)	1,120 (3,440; 14,400)	177.0 (88.00; 314.00)	7.3 (2; 47.90)
Other pathogens (6)	7 (1; 15)	3,083 (1,835; 5,998)	218.5 (174.00; 317.80)	4.1 (0; 29.25)
Unknown etiology (24)	22 (6; 46)	505 (159; 1490)	78.20 (35.00; 1350.00)	51,60 (45.10; 70.00)

Values are represented as medians (25; 75 percentiles)

### Sample collection and processing

Blood samples were collected and processed by centrifugation at 2,880 g and 4 °C for 3 min to separate the plasma. Genomic DNA extraction was performed using the salting out procedure following the protocol described by Miller *et al*. [23]. Samples of CSF were collected upon lumbar puncture (LP) and centrifuged at 720 g for 5 min. Supernatants were frozen and stored at −80 °C before any further procedure. Blood samples were obtained from all patients and volunteers. However, given that LP is a very invasive procedure, CSF samples were obtained only from patients undergoing procedures for the diagnosis of meningitis. Patients who had a positive diagnosis were included in the BM group.

### Genotypic analysis

The SNPs investigated in this study are detailed in Table 2. The primer sequences for *TNF* -308G > A, *TNF* -857C > T, and *IL8* -251A > T have been previously described [24–26]. Primer-introduced restriction analysis-polymerase chain reaction (PIRA-PCR) was carried out to identify the SNP *TNF* -308G > A, containing a single-base mismatch leading to the amplification of PCR products containing a restriction site. For all SNPs, genotyping was performed following the digestion of the amplified fragments with appropriate restriction endonucleases using the methodology for restriction fragment length polymorphism (PCR-RFLP) (Table 3). PCR was carried out using 100 ng of genomic DNA in a 25-μl volume reaction containing 1X specific buffer, 10 pM of each primer, 2.0 mM MgCl2, 0.3 mMol of each dNTP, and 3.6 U of Taq polymerase. The PCR conditions were as follows: initial denaturation at 94 °C for 5 min, followed by 35 cycles of 45 s at 94 °C for denaturation and annealing for 45 s (temperatures given in Table 3), elongation at 72 °C for 1 min, and a final extension step at 72 °C for 5 min. PCR products were run on 2 % agarose gels and visualized with ethidium bromide. The digestion of PCR products was performed following the instructions of the manufacturers of the restriction enzymes. The digestion products were analyzed on an 8 % polyacrylamide gel and were revealed with silver staining according to the protocol described by Sanguinetti *et al*. [27]. Quality control was carried out with 20 % repetition of the entire sample for each SNP to evaluate data reproducibility.

**Table 2:** Polymorphisms analyzed in this study

Polymorphism	Reference	Chromosome	Functional position	Wild type/rare allele
*TNF* -308G > A	rs1800629	6	promoter region	G/A
*TNF* -857C > T	rs1799724	6	promoter region	C/T
*IL8* -251A > T	rs4073	4	promoter region	A/T
*APEX1* Asn148Glu	rs1130409	14	exon	G/T
*OGG1* Ser326Cys	rs1052133	3	exon	C/G
*PARP1* Val762Ala	rs1136410	1	exon	T/C
*AADAT* +401C > T	rs1480544	4	splice site	C/T

**Table 3:** PCR conditions for genotyping: primer sequences, annealing temperatures and restrictions enzymes

SNP	Primer sequence	Annealing temperature	Restriction enzyme
*TNF* -308G > A	F 5’AGGCAATAGGTTTTGAGGGCCAT'3	55 °C	*Nco*I
	R 5’TCCTCCCTGCTCCGATTCCG'3		
*TNF* -857C > T	F 5’GGCTCTGAGGAATGGGTTAC'3	58 °C	*Tai*I
	R 5’CCTCTACATGGCCCTGTCTAC'3		
*IL8* -251A > T	F 5’CCATCATGATAGCATCTGTA'3	53 °C	*Ase*I
	R 5’CCACAATTTGGTGAATTATTAA'3		

### Cytokine multiplex measurements in CSF and plasma

Proinflammatory cytokine and chemokine levels in the CSF and plasma were measured during the acute phase of disease and before the start of treatment with antibiotics. Analysis was performed with microsphere-based multiplex assays (xMAP Luminex technology) using the Bio-Plex 200 suspension array system (Bio-Rad, Hercules, CA, USA). A human cytokine Lincoplex Kit (HCYTO-60 k, Millipore, Billerica, Ma, USA) was used to analyze a set of 12 cytokines and chemokines (TNF-α, IL-6, IL-1β, INF-γ, IL-2, IL-10, IL-1RA, MIP-1α/CCL3, MIP-1β/CCL4, MCP-1/CCL2, G-CSF and IL-8/CXCL8). The samples were processed and measured according to the manufacturer’s instructions, as described previously [10, 12, 13]. Cyto/chemokine expression was measured in duplicate, and the inflammatory modulators levels were analyzed in relation to a parametric logistic curve using Bio-Plex manager 4.01 software and expressed as pg/ml. Due to the low quantities of biological material, evaluation of all patients was not possible.

### Statistical analysis

Allelic and genotypic frequencies were calculated for patient and control subjects via direct gene counting. Hardy-Weinberg equilibrium was evaluated using GENEPOP 4.2 software (http://genepop.curtin.edu.au/). For linkage disequilibrium analysis (LD) of the *TNF* -308G > A and *TNF* -857C > T polymorphisms, Haploview software version 4.2 was used as described previous [28]. Logistic regression analyses were used to calculate odds ratios (OR), 95 % confidence intervals (CI) and corresponding *P-*values to determine the association between each variant and susceptibility to BM. In this analysis, the effects of age and sex were evaluated. STATA software (version 11.0; Stat Corporation, College Station, Texas, USA) was used to conduct this statistical analysis. The generalized multifactor dimensionality reduction (GMDR) analysis was used to assess gene–gene interactions with GMDR Beta 0.9 software. This method has been shown to be effective for detecting gene-gene interactions in case–control studies with relatively small sample sizes. The best statistical gene–gene interactions model for a given order of interaction was determined by three factors: the cross-validation consistency statistics for the selected SNP combinations, the prediction accuracies and the significance level or *P*-value, for which all possible interactions were tested 10 times in an exhaustive search [29, 30]. A total of 1,000 permutation tests were performed in each analysis. For statistical analysis, we used the autosomal dominant model (Aa + aa vs. AA) wherein the presence of the “a” allele is sufficient to show the phenotype. In addition, differences in levels of inflammatory modulators were analyzed using GraphPad Prism5 software. It was used the non-parametric Mann–Whitney *U* test, for comparisons between two groups, and Kruskal-Wallys test followed by Dunn's Multiple Comparison post-test. For the correlation analysis, it was used the Spearman test.. Values of *P <* 0.05 (two-tailed) were considered statistically significant.

## Results

### Genotypic frequency analysis

The distribution of the genotypic frequencies of the *TNF* -308G > A, *TNF* -857C > T, and *IL8* -251A > T polymorphisms in BM patients and controls are shown in Table 4. All genotypic distributions were in Hardy-Weinberg equilibrium (Additional file 1: Table S1). Haplotype analysis indicated a D’ value of 0.11 for the *TNF* polymorphisms -308G > A and -857C > T, suggesting that they segregate independently (Additional file 1: Figure S1). No significant differences between the BM and control groups were found in relation to the SNPs *TNF* -857C > T and *IL8* -251A > T. However, in the multivariate regression model, an association between the *SNP* TNF -308G > A and a possible protective role against BM was observed; even after adjustment for age and sex, the comparison remained statistically significant (*P =* 0.020 and *P* adjusted = 0.030). Individuals carrying the GA genotype demonstrated a significant reduction for BM risk compared to those carrying the GG genotype (OR = 0.292 and 95 % CI = 0.12–0.711). Individuals carrying the AA genotype had an OR of 0.375 compared with carriers of the GG genotype (95 % CI = 0.041–0.711). The frequency of the variant allele was decreased in the BM group (0.08) compared to the control group (0.18). Of a total of 163 individuals genotyped for the SNP *TNF* -308G > A, 70 % were homozygous for the wild type allele, 26 % were heterozygous, and 4 % were homozygous for the variant allele.

**Table 4:** Genotypic frequencies of the SNPs for BM compared with the control group

Genotypes and alleles	Control group n (%)	BM group n (%)	*P for difference*	OR (95 % CI)	Adjusted *P for difference*^a^	Adjusted OR (95 % CI) ^a^
*TNF −308*	*(controls = 109; cases = 54)*					
GG	69 (63)	46 (85)	0.020*	1	0.030*	1
GA	36 (33)	7 (13)		0.292 (0.12-0.711)		0.319 (0.129-0.790)
AA	4 (4)	1 (2)		0.375 (0.041-0.711)		0.311 (0.033-2.943)
*TNF −*857	*(controls = 107; cases = 52)*					
CC	81 (76)	39 (75)	0.92	1	0.92	1
CT	26 (24)	13 (25)		1.038 (0.482-2.237)		1.475 (0.475-2.290)
TT	0 (0)	0 (0)				
*IL8* -251	*(controls = 106; cases = 52)*					
AA	31 (29)	12 (23)	0.62	1	0.68	1
AT	43 (41)	25 (48)		1.502 (0.656-3.44)		1.398 (0.596-3.280)
TT	32 (30)	15 (29)		1.211 (0.490-3.00)		1.058 (0.416-2.690)

*BM* bacterial meningitis, *OR* odds ratio, *CI* confidence interval. Data analyzed by multivariate logistic regression analyses. ^*^Statistical significance (*P* < 0.05). The reference group in each of the analyses was the most prevalent genotype. ^a^Data adjusted for age and gender

The individual allelic and genotypic frequencies for the SNPs *AADAT* +401C > T, *APEX1* Asn148Glu, *OGG1* Ser326Cys and *PARP1* Val762Ala were previously published [12, 13].

### Gene-gene interaction analysis

To identify possible combinations of genotypes that could affect the occurrence of the disease, we performed an analysis of gene-gene interactions for each polymorphism. According to the GMDR data, four different combination models were obtained, which suggested the best models for the combination of all SNPs that exhibited associations with BM (Table 5). We determined the best interaction models based on a cross-validation of >7 of 10, a prediction accuracy >55 % and a *P*-value <0.05 for each model. The combinations of *APEX1* Glu/_, *AADAT* T/_ (*P* = 0.0107), *IL8* T/_, *APEX1* Glu/_, *AADAT* T/_ (*P* = 0.0010), *IL8* T/_, *APEX1* Glu/_, *OGG1* Cys/_, *PARP1* Ala/_, and *AADAT* T/_ (*P* = 0.0107) were statistically significant, suggesting the variants together may contribute to the occurrence of disease. The combination of the three variant alleles of the DNA repair enzymes was also more frequent in the BM group, although it was not statistically significant (*P =* 0.0547), but the best combination was the two-locus model with *APEX1* Glu/_ and *OGG1* Cys/_ (*P =* 0.0107). Regarding phenotypes that conferred susceptibility to BM, the best gene-gene interaction model was the wild type genotype of *TNF* -308G/G with *APEX1* Glu/, *IL8* -251 T/_ and *AADAT* +401 T/_ (*P =* 0.0107).

**Table 5:** GMDR results of multi-locus interaction with BM compared with the control group

Best model (All susceptibility SNPs)	Testing balanced accuracy	Sign test (*P-*value)	Cross-validation accuracy
*AADAT* T/_	0.6305	9 (0.0107)^*^	9/10
*APEX1* Glu/_, *AADAT T/_*	0.6359	9 (0.0107)^*^	9/10
*IL8* T/_,*APEX1* Glu/_, *AADAT T/_*	0.6586	10 (0.0010)^*^	10/10
*IL8* T/_,*APEX1* Glu/_, *OGG1* Cys/_, *AADAT T/_*	0.5743	6 (0.3770)	6/10
*IL8* T/_,*APEX1* Glu/_, *OGG1* Cys/_, *PARP1* Ala/_,*AADAT T/_*	0.6302	9 (0.0107)^*^	10/10
Best model (DNA repair enzymes)	Testing balanced accuracy	Sign test (*P-*value)	Cross-validation accuracy
*APEX1* Glu/_	0.6605	9 (0.0107)^*^	10/10
*APEX1* Glu/_, *OGG1* Cys/_,	0.5850	9 (0.0107)^*^	8/10
*APEX1* Glu/_, *OGG1* Cys/_, *PARP1* Ala/_,	0.6510	8 (0.0547)	10/10
Best model (protection SNPs)	Testing balanced accuracy	Sign test (*P-*value)	Cross-validation accuracy
*TNF-308 A/_*	0.5939	9 (0.0107)^*^	10/10
*TNF-308 A/_, TNF-857 T_*	0.5758	8 (0.0547)	10/10
Best model (susceptibility alleles)	Testing balanced accuracy	Sign test (*P-*value)	Cross-validation accuracy
*TNF-308 GG, AADAT T/_*	0.6267	8 (0.0547)	7/10
*TNF-308 GG, AADAT T/_, APEX1* Glu/_,*IL8* T/_,	0.6108	9 (0.0107)^*^	7/10
*TNF-308 GG, AADAT T/_, APEX1* Glu/_, *IL8* T/_, *OGG1* Cys/_	0.5528	7 (0.1719)	5/10
*TNF-308 GG, AADAT T/_, APEX1* Glu/_, *IL8* T/_, *OGG1* Cys/_, *PARP1* Ala/_,	0.5553	7 (0.1719)	10/10

^*^Significant *P-*value (*P <* 0.05); *P-*value was based on 1,000 permutations. Dominant inheritance model (Aa + aa vs. AA)

In addition, although isolated effects of the SNP *TNF* -857C > T were not observed, the combination of both variants of the *TNF* gene resulted in marginal significance (*P* = 0.0547) in terms of the protection against the disease. Together, the data obtained reveal possible associations between different genes that may interact with each other, leading to the occurrence of BM.

### Association of SNPs with immune response markers

**Fig. 1:**
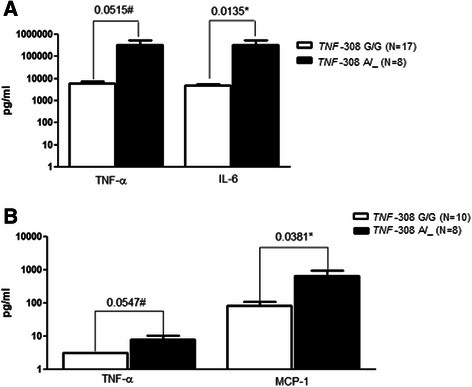
Cytokine and chemokine concentrations in relation to the genotype for SNP *TNF* -308G > A. **a** CSF samples of BM patients. **b** Plasma samples of controls. Legend: White Bar - cytokine levels for patients with ancestral genotype; Black bar - cytokine levels for patients with at least one polymorphic allele. *Statistical significance (*P* < 0.05); # borderline values

In the analysis of cyto/chemokines in relation to individual genotypes, we observed some significant changes between the BM and control groups for the SNP *TNF* -308G > A alone. An increase in TNF-α and IL-6 levels was observed in CSF samples from BM patients carrying the polymorphic *TNF* -308A allele (Fig. 1a), while an increase in TNF-α and MCP-1 was observed in plasma samples from the control group (Fig. 1b). The SNP *TNF* -308G > A did not exert an effect on other inflammatory modulators (Additional file 1: Figure S2). For the SNPs *TNF* - 857C > T and *IL8* -251A > T, no significant difference in the levels of immune markers was observed.

**Fig. 2:**
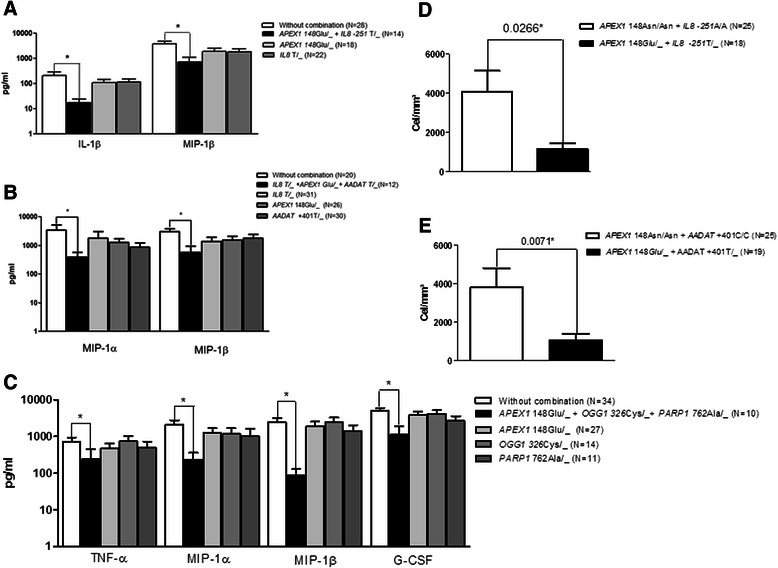
Cyto/chemokine concentrations and cell counts in CSF in relation to the combination of genotypes in BM patients. **a** *APEX1* 148Glu/_ plus *IL8* -251 T/_ combination. **b** *IL8* -251 T/_ plus *APEX1* 148 Glu/_ and *AADAT* +401 T/_ combination. **c** *APEX1* 148Glu/_ plus *OGG1* 326Cys/_ and *PARP1* l762Ala/_ combination. **d** Cell counts associated with the combined genotype *APEX1* 148Glu/_ plus *IL8* -251 T/_. **e** Cell counts associated with the combined genotype *APEX1* 148Glu/_ plus *AADAT* +401 T/_. *Statistical significance (*P <* 0.05)

Synergism between the SNPs was also investigated. Patients carrying the combination of *APEX1* 148Glu/_ plus *IL8* -251 T/_ exhibited reduced levels (*P <* 0.05) of IL-1β and MIP-1β/CCL4 (Fig. 2a). The combination of *IL8* T/_ plus *APEX1* Glu/_ plus *AADAT* T/_ also induced the reduction of cytokines TNF-α, MIP-1α/CCL3, and MIP-1β/CCL4 (Fig. 2b) in CSF samples from BM patients. In presence of the three variant alleles of the DNA repair genes, a decrease (*P <* 0.05) in the levels of TNF-α, MIP-1α/CCL3, MIP-1β/CCL4, and G-CSF in the CSF (Fig. 2c) was observed. The other combinations did not demonstrate significant changes in the concentrations of cyto/chemokines (Additional file 1: Figures S3 and S4).

The relationships between the levels of these inflammatory modulators and cell counts in the CSF of BM patients were also assessed in terms of genotypic combinations. A significant reduction in cell count was observed in patients with the combined genotype *APEX1* 148Glu/_ plus *IL8* -251T/_ or *APEX1* 148Glu/_ plus *AADAT* +401T/_ (Figs. 2d and e). Analysis of these combinations demonstrated significant correlations between cell count and IL-1β, IL-6, TNF-α, MIP-1α/CCL3, MIP- 1β/CCL4 and G-CSF (Table 6). These data suggest a possible synergism between the polymorphisms, given that an additive effect in terms of reduced levels of inflammatory modulators was observed. Other genotypic combinations did not achieve significance in terms of cell count (Additional file 1: Figure S5).

**Table 6:** Correlation between cell count and cytokine and chemokine levels in CSF from BM patients in terms of genotypic combinations

Genotypic combinations	Cytokines and chemokines						
*APEX1* + 148 Asn/Asn plus *IL8* -251 AA	IL-1β	IL-6	TNF-α	MIP1-α	MIP1-β	MCP-1	G-CSF
Spearman r	0.428	0.305	0.391	0.204	0.422	−0.155	−0.037
*P-*value	0.047^*^	0.168	0.072	0.363	0.050^*^	0.490	0.870
*APEX1* + 48Glu/_ plus *IL8* -251 T/_	IL-1β	IL-6	TNF-α	MIP1-α	MIP1-β	MCP-1	G-CSF
Spearman r	0.830	0.547	0.781	0.735	0.791	0.346	0.788
*P-*value	<0.001^*^	0.019^*^	0.0013^*^	<0.001^*^	<0.001^*^	0.159	<0.001^*^
*APEX1* + 148 Asn/Asn plus *AADAT* + 401 CC	IL-1β	IL-6	TNF-α	MIP1-α	MIP1-β	MCP-1	G-CSF
Spearman r	0.111	0.506	0.311	−0.100	0.032	−0.007	−0.104
*P-*value	0.673	0.038^*^	0.224	0.701	0.903	0.978	0.691
*APEX1* + 148 Glu/_ plus *AADAT* +401 T/_	IL-1β	IL-6	TNF-α	MIP1-α	MIP1-β	MCP-1	G-CSF
Spearman r	0.783	0.623	0.579	0.507	0.645	0.060	0.625
*P-*value	<0.001^*^	0.007^*^	0.005^*^	0.012^*^	0.005^*^	0.818	0.007^*^

^*^Significant *P-*value (*P <* 0.05). Correlation analysis was performed using a two-tailed Spearman test (cell count vs. cytokine or chemokine levels)

## Discussion

The genetic characteristics of patients are considered important factors in BM occurrence [2–4]. In a previous study, we identified the association of the variant alleles *AADAT* +401C > T and *APEX1* Asn148Glu with the occurrence of BM. These SNPs were also shown to influence both the innate and adaptive immune responses [12, 13]. In the present work, we analyzed the association of SNPs in the *TNF* and *IL-8* genes and investigated the effects of combining all SNPs, including previously published SNPs [12, 13]. The low frequency of disease and the poor public health system in Natal (Brazil) did not permit the recruitment of a large group of patients for the study, but our sample size is in accordance with the epidemiological and clinical spectrum of BM in our region [31, 32].

Systematic differences in ancestry between cases and controls can be found when genetically distinct subgroups demonstrate differing prevalences of target phenotypes [33]. When heterogeneity is equivalent in cases and controls (i.e., the two groups have the same mixture of ethnic/genetic subgroups), stratification does not occur, such as in admixed populations [34]. Although we did not investigate ancestry in our samples, it is known that the Brazilian population has great potential for research due to its admixture, and the various combinations of variants at different loci facilitates the study of gene-gene interactions, which in other homogeneous populations would not be possible. In our specific population, samples were obtained from individuals from northeastern Brazil, which has no specific ethnicity; indeed, this population has the highest degree of admixture in Brazil [35].

Significant differences in the distribution of the individual *TNF* -857C > T and *IL8* -251A > T variant genotypes between patients and healthy controls were not found (Table 4). However, for the SNP *TNF* -308G > A, a higher prevalence of the heterozygote in the control group and the homozygote for the wild type allele in the BM group (Table 4) was observed, suggesting a possible protective role against the disease, which must be further investigated. The genotypic frequencies found in our work for SNP *TNF* -308G > A are similar to those found in other populations [36–38].

Alterations in cytokine and chemokine profiles were observed in the presence of the *TNF* -308A allele (Fig. 1). Despite the fact that the *P-*value for TNF-α levels had a borderline value, the biological relevance may be considered, as statistical significance was obtained for other cytokines regulated by TNF-α. These data corroborate previous work that reported the effect of the SNP *TNF* -308G > A on *TNF* gene transcription [16, 17]. TNF-α is one of the primary pro-inflammatory cytokines that plays a central role in initiating and regulating the inflammatory response, acting as a mediator of resistance to infectious agents, and therefore is important in host defense. TNF-α is involved in the signal transduction pathways that activate cellular NF-κB, which is a transcription factor that regulates the expression of many pro-inflammatory genes [39–41]. Furthermore, TNF-α is indirectly related to the production of immunoglobulin via TGF-β1 induction [42].

In several infection models, an inefficient immune response by cytokines and chemokines was associated with a decrease in the clearance of pathogens, the development of invasive disease and high mortality [43–45]. Low levels of IL-1β, TNF-α or IL-6 in nasopharyngeal secretions were observed in children with recurrent episodes of acute otitis media, an important cause of meningitis [46]. These cytokines are important for the activation of chemokines (such as MIP-1α/CCL3 and MIP-1β/CCL4) involved in leukocyte recruitment to the infection site for effective pathogen eradication [47]. However, the severity of meningococcal meningitis is directly correlated with the production of IL-1β, TNF-α, IL-6 and IL-8 [48, 49].

For complex diseases, the effect of an individual gene is likely to be small and may be modulated by gene-gene or gene-environment interactions [50]. Considering that the pathophysiology of infectious disease involves the interaction of several proteins and pathways, certain genetic combinations suggest a risk factor for the occurrence of severe BM [3, 4]. In this work, certain genotypic combinations were prevalent in BM patients. After analyzing the genotype distributions of the *SNP* combinations between cases and controls, we found that there were five genotypic combinations that exhibited the highest frequencies in the BM patient group compared to the control group, particularly the *IL-8*T/_, *APEX1* Glu/_, *OGG1* Cys/_, *PARP1* Ala/_, and *AADAT T/_* genotypes (Table 5). This indicated that interaction of these five *SNPs* may result in the occurrence of BM. The combination of the wild type genotype of the TNF gene (GG) with the variant alleles *AADAT* T/_, *APEX1* Glu/_, and *IL8* T/_ also resulted in statistical significance.

In contrast to the other SNPs analyzed, the combination of the two variant alleles of the *TNF* gene exhibited an interesting additive effect in the control group. Individually, the SNP *TNF* -857C > T frequency did not suggest an association with BM, as there were no differences between BM and the control group. However, in the control group, the frequency of both the variant *TNF -*857T and *TNF -*308A alleles was higher than the BM group, suggesting a possible protective role against infection. However, further studies are needed to confirm this hypothesis.

Analysis of the combined effects of SNPs in comparison to the levels of inflammatory modulators also demonstrated significant associations that may contribute to the increased risk for BM occurrence. A synergistic effect in cytokine and chemokine expression was observed for some genetic combinations. The SNPs *APEX1* Asn148Glu, *PARP1* Val762Ala and *OGG1* Ser326Cys were previously described to be involved in the reduction of DNA repair function [13, 51–53]. In this study, the presence of variant alleles of DNA repair genes was associated with a reduction in the levels of TNF-α, MIP-1α/CCL3, MIP-1β/CCL4 and G-CSF, suggesting a less active inflammatory response in patients who possess this combination.

APE1 and PARP-1 were initially known as DNA repair enzymes but are also important activators and co-factors of the DNA-binding activity of NF-κB and AP-1 (activator protein1) [54–59], which are master regulators of the inflammatory response [41, 59]. In addition, these two enzymes may contribute to the regulation of humoral immunity by participating in immunoglobulin class switch recombination [60, 61].

In inflammation models such as endotoxic shock, diabetes and contact hypersensitivity, *OGG1* gene knockout in mice was shown to be associated with decreased serum cytokine and chemokine levels, which highlights the importance of this enzyme in the inflammatory response [62].

In addition to the role of DNA repair enzymes in the activation of NF-κB and AP-1, some work has proposed a role for the BER pathway in transcriptional activation, although the mechanisms involved are not well understood. The occurrence of oxidized bases in promoter regions may affect the binding properties of transcriptional factors such as AP-1 [63] or interfere with CpG island methylation, an event that commonly suppresses gene transcription [64]. Recently, Khobta *et al*. [65] obtained data showing that oxidized bases induce gene silencing in a potential chromatin-mediated mechanism associated with a decrease in histone H4 acetylation in the promoter region. In addition, the demethylation activity mediated by the LSD1 enzyme produces H_2_O_2_, which locally oxidizes guanine and induces the recruitment of OGG1 and APE1, which are involved in the chromatin remodeling that is necessary for transcriptional activation of the target genes [66]. Based on these studies, polymorphisms in DNA repair enzymes may generate aberrant gene expression, triggering changes in the inflammatory response.

IL-8/CXCL8 is an important mediator that has been implicated in biochemical pathways involved in a wide range of inflammatory diseases [19–21, 67–69]. Activation of CXCL8/IL8 in gastric epithelial cells infected with *H. pylori* is directly dependent on the activation of AP-1, which is regulated by APE1. The silencing of APE1 expression by siRNA causes a reduction in *H. pylori*-induced IL-8 mRNA and protein, supporting the hypothesis that APE1 is involved in the control of gene expression in bacterial pathogenesis [69]. Although the SNP *IL8* -251A > T was not related to BM occurrence, combination with the variant allele *APEX1* 148Glu resulted in statistical significance (Table 5). The analysis of immune markers provides evidence that these polymorphisms may exhibit synergism, as a reduction in cytokine and chemokine levels and in cell counts was observed (Fig. 2).

Activation of the KYN pathway was observed during the acute phase of pneumococcal meningitis in infant rats [70]. In a previous study, we also found an association between the kynurenine aminotransferase II gene C401T polymorphism (SNP *AADAT* +401C > T) and BM occurrence [12]. Recently, we observed that this polymorphism is associated with an increase in kynurenic acid (KYNA) levels [22], which has anti-inflammatory effects, as it is involved in modulating the immune responses mediated by activation of G protein-coupled receptor 35 (GPR35) [71]. This may explain the lower levels of cyto/chemokines and cell counts observed in patients carrying the T allele.

The role of chemokines in regulating leukocyte recruitment during infection has also been reported [45, 72, 73]. We observed a significant correlation (*P* < 0.05) between cell counts and IL-1β, IL-6, TNF-α, MIP1-α, MIP1-β and G-CSF levels in BM patients who had genotypic combinations involving the alleles *APEX1* 148Glu, *IL8* -251T and *AADAT* +401T (Table 6), suggesting a reduced capacity for leukocyte recruitment in these patients.

During the BM acute phase, an increase occurs in the expression of some cytokines, such as TNF-α and IL-6, which is useful for differential diagnosis with regard to other meningitis types such as aseptic or chronic meningitis [74–76]. As our biological samples intended for the measurement of inflammatory modulators were collected in the acute phase of the disease and the results of the genotype comparisons with reference to cytokine and chemokine levels were not significantly influenced by the time of collection.

## Conclusions

In conclusion, analysis of individual polymorphisms suggested that the *TNF* -308A allele may play a potential role in protecting against BM. In addition, genetic combinations, primarily involving the *TNF* -308G, *APEX1* 148Glu, *IL8* -251T and *AADAT* +401T alleles, were shown to be associated with the inflammatory process in general, leading to decreased expression of cyto/chemokines. This may be related to a reduced ability to eradicate the pathogen and consequent susceptibility to disease. In this paper, we proposed a possible model for gene-gene interactions between important pathways that are activated during BM. However, the low number of patients is a limitation of our work; therefore, collaborative studies conducted in different populations are necessary to corroborate our findings. Our data indicate that an extensive evaluation of the variability of these genes may contribute to a better understanding of susceptibility to BM and potentially to other infectious or inflammatory diseases. This knowledge will not only permit clinical interventions but also the implementation of programs for health promotion and disease prevention directed towards susceptible individuals based on their genomic profile, which may lead to decreased morbidity and mortality through risk assessment and improvement of the administration of prophylactic therapies.

## Additional file

Additional file 1: Table S1. Allelic and genotypic frequencies of the SNPs in the studied population. **Figure S1.** Haplotype map showing the two TNF analyzed polymorphism and a D’ value of 0.11, suggesting that the TNF polymorphisms segregate independently (linkage equilibrium). **Figure S2.** Cytokine and chemokine concentration in relation to the genotype for SNP *TNF* -308G>A. (A): CSF samples of BM patients. (B): Plasma samples of controls. **Figure S3.** Cyto/chemokine concentration in CSF samples in relation to the combination of genotypes in BM patients. (A): *APEX1* 148Glu/_ plus*IL8* -251 T/_ combination. (B): *APEX1* 148Glu/_ plus *OGG1* 326Cys/_ and *PARP1* l762Ala/_ combination. (C): *IL8* T/_ plus *APEX1*Glu/_ and *AADATT/_*combination. (D): *APEX1* 148 Glu/_ plus *AADAT* +401 T/_ combination. (E): *APEX1* 148Glu/_ and *OGG1* 326Cys/_ combination. (F): *TNF −*308 GG plus*IL8* T/_ plus *APEX1*Glu/_ and *AADATT/_* combination. **Figure S4.** Cyto/chemokine concentration in Plasma samples in relation to the combination of genotypes in controls. (A): *APEX1* 148Glu/_ plus*IL8* -251 T/_ combination. (B): *APEX1* 148 Glu/_ plus *AADAT* +401 T/_ combination. (C): *IL8* T/_ plus *APEX1*Glu/_ and *AADATT/_* combination. It was not possible to compare all combinations in this biological sample due to the small amount of material. **Figure S5.** Cell count in CSF samples in relation to the combination of genotypes in BM patients: (A): *APEX1* 148Glu/_ plus *OGG1* 326Cys/_ and *PARP1* l762Ala/_ combination. (B): *IL8* T/_ plus *APEX1* Glu/_ and *AADATT/_* combination. (C): *APEX1* 148Glu/_ and*OGG1* 326Cys/_ combination. (D): *TNF −*308 GG plus*IL8* T/_ plus *APEX1*Glu/_ and *AADATT/_* combination. (DOCX 773 kb)

## Abbreviations

AADAT: Aminoadipate aminotransferase
AP-1: Activator protein 1
APE1: Apurinic/apyrimidinic endonuclease 1
BER: Base excision repair
BM: Bacterial meningitis
CNS: Central nervous system
CSF: Cerebrospinal fluid
GMDR: Multifactor dimensionality reduction
GPR35: G protein-coupled receptor 35
HGT: Hospital Giselda Trigueiro
Hib: *Haemophilus influenzae* type b
KYN: Kynurenine
KYNA: Kynurenic acid
LP: Lumbar puncture
MMPs: Matrix metalloproteinases
NF-κB: Nuclear transcription factor kappa B
OGG1: 8-oxoguanine DNA glycosylase-1
PARP-1: Poly(ADP-ribose) polymerase-1
PCR-RFLP: Polymerase chain reaction-restriction fragment length polymorphism
PIRA-PCR: Primer-introduced restriction analysis-polymerase chain reaction
RNI: Reactive nitrogen intermediates
ROS: Reactive oxygen species
SNP: Single nucleotide polymorphisms
TLRs: Toll-like receptors
TNF-α: Tumor necrosis factor
