# Supplementary material for: Genetic polymorphisms associated with the inflammatory response in bacterial meningitis
Source: BMC Med Genet. 2015 Aug 28;16:70. doi: 10.1186/s12881-015-0218-6 (PMC4593216; doi:10.1186/s12881-015-0218-6)
Supplement: Additional file 1: Table S1. — Allelic and genotypic frequencies of the SNPs in the studied population. Figure S1. Haplotype map showing the two TNF analyzed polymorphism and a D’ value of 0.11, suggesting that the TNF polymorphisms segregate independently (linkage equilibrium). Figure S2. Cytokine and chemokine concentration in relation to the genotype for SNP TNF -308G>A. (A): CSF samples of BM patients. (B): Plasma samples of controls. Figure S3. Cyto/chemokine concentration in CSF samples in relation to the combination of genotypes in BM patients. (A): APEX1 148Glu/_ plusIL8 -251 T/_ combination. (B): APEX1 148Glu/_ plus OGG1 326Cys/_ and PARP1 l762Ala/_ combination. (C): IL8 T/_ plus APEX1Glu/_ and AADATT/_combination. (D): APEX1 148 Glu/_ plus AADAT +401 T/_ combination. (E): APEX1 148Glu/_ and OGG1 326Cys/_ combination. (F): TNF −308 GG plusIL8 T/_ plus APEX1Glu/_ and AADATT/_ combination. Figure S4. Cyto/chemokine concentration in Plasma samples in relation to the combination of genotypes in controls. (A): APEX1 148Glu/_ plusIL8 -251 T/_ combination. (B): APEX1 148 Glu/_ plus AADAT +401 T/_ combination. (C): IL8 T/_ plus APEX1Glu/_ and AADATT/_ combination. It was not possible to compare all combinations in this biological sample due to the small amount of material. Figure S5. Cell count in CSF samples in relation to the combination of genotypes in BM patients: (A): APEX1 148Glu/_ plus OGG1 326Cys/_ and PARP1 l762Ala/_ combination. (B): IL8 T/_ plus APEX1 Glu/_ and AADATT/_ combination. (C): APEX1 148Glu/_ andOGG1 326Cys/_ combination. (D): TNF −308 GG plusIL8 T/_ plus APEX1Glu/_ and AADATT/_ combination. (DOCX 773 kb) [file 12881_2015_218_MOESM1_ESM.docx]

Additional file 1

Table S1: Allelic and genotypic frequencies of the SNPs in the studied population.

| SNP | *p* | *q* | Observed genotypic frequency | | | *P*-value^a^ |
| --- | --- | --- | --- | --- | --- | --- |
|  |  |  | *p*^2^ | 2*pq* | *q*^2^ |  |
| *TNF* -308G>A | 0.836 | 0.164 | 0.70 | 0.27 | 0.03 | 0.7713 |
| *TNF* -857C>T | 0.879 | 0.121 | 0.76 | 0.24 | 0.0 | 0.1295 |
| *IL8* -251A>T | 0.487 | 0.513 | 0.27 | 0.43 | 0.30 | 0.0817 |
| *APEX1* Asn148Glu | 0.701 | 0.299 | 0.50 | 0.40 | 0.10 | 0.5714 |
| *OGG1* Ser326Cys | 0.779 | 0.221 | 0.60 | 0.35 | 0.05 | 1.0000 |
| *PARP1* Val762Ala | 0.837 | 0.163 | 0.70 | 0.29 | 0.01 | 0.5779 |
| *AADAT* +401C/T | 0.634 | 0.366 | 0.42 | 0.44 | 0.14 | 0.4104 |

- The Hardy-Weinberg equilibrium was evaluated in the total population (163 individuals, BM + control). ^a^*P*-value of the Chi square test. All SNPs are in Hardy-Weinberg equilibrium


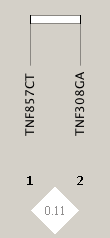


Figure S1: Haplotype map showing the two TNF analyzed polymorphism and a D’ value of 0.11, suggesting that the TNF polymorphisms segregate independently (linkage equilibrium).

**Figure S2**

A

B

**Figure S2:** Cytokine and chemokine concentration in relation to the genotype for SNP *TNF* -308G>A. (A): CSF samples of BM patients. (B): Plasma samples of controls.

**Figure S3**

A

B

C

D

E

F

**Figure S3:** Cyto/chemokine concentration in CSF samples in relation to the combination of genotypes in BM patients. (A): *APEX1* 148Glu/_ plus*IL8* -251 T/_ combination. (B): *APEX1* 148Glu/_ plus *OGG1* 326Cys/_ and *PARP1* l762Ala/_ combination. (C): *IL8* T/_ plus *APEX1*Glu/_ and *AADATT/_*combination. (D): *APEX1* 148 Glu/_ plus *AADAT* +401 T/_ combination. (E): *APEX1* 148Glu/_ and *OGG1* 326Cys/_ combination. (F): *TNF* -308 GG plus*IL8* T/_ plus *APEX1*Glu/_ and *AADATT/_* combination.

**Figure S4**

A

B

C

**Figure S4:** Cyto/chemokine concentration in Plasma samples in relation to the combination of genotypes in controls. (A): *APEX1* 148Glu/_ plus*IL8* -251 T/_ combination. (B): *APEX1* 148 Glu/_ plus *AADAT* +401 T/_ combination. (C): *IL8* T/_ plus *APEX1*Glu/_ and *AADATT/_* combination. It was not possible to compare all combinations in this biological sample due to the small amount of material.

**Figure S5**

A

B

C

D

**Figure S5:**  Cell count in CSF samples in relation to the combination of genotypes in BM patients: (A): *APEX1* 148Glu/_ plus *OGG1* 326Cys/_ and *PARP1* l762Ala/_ combination. (B): *IL8* T/_ plus *APEX1* Glu/_ and *AADATT/_* combination. (C): *APEX1* 148Glu/_ and*OGG1* 326Cys/_ combination. (D): *TNF* -308 GG plus*IL8* T/_ plus *APEX1*Glu/_ and *AADATT/_* combination.
